# Exclusion of introduced deer increases size and seed production success in an island‐endemic plant species

**DOI:** 10.1002/ece3.1885

**Published:** 2016-01-09

**Authors:** Tyler M. Dvorak, Amy E. Catalano

**Affiliations:** ^1^ Conservation DepartmentCatalina Island ConservancyP.O. Box 2739AvalonCalifornia90704

**Keywords:** *Crocanthemum greenei*, deer, exclosure, herbivory, invasion, island

## Abstract

The presence of extra‐local invaders, such as the southern California mule deer (*Odocoileus hemionus*) on Santa Catalina Island, may contribute to more selective and insidious effects within the unique ecosystems that have evolved in their absence. Studies at the species level may detect effects not noticed in broader, community level vegetation monitoring or help tease apart differences in the level of effect among the various ecological components of an invaded system. In this initial study, we measured the impacts of herbivory by mule deer, a species native to analogous habitats on the adjacent mainland, on size and seed production success for *Crocanthemum greenei* (island rush‐rose), a federally listed sub‐shrub that is not present on mainland California. We found deer exclusion resulted in an overall increase in stem measurement of 18.8 cm. Exclosure populations exhibited complete seed production success, whereas control populations showed significantly reduced success and exhibited complete failure within 58% of populations. These results show that the introduced mule deer on Santa Catalina Island are negatively affecting a federally threatened plant species. This strongly implies that the current deer management strategy is insufficient, if one of its goals is biodiversity and endemic species conservation.

## Introduction

The negative impacts of introduced ungulates on island flora and ecosystems, particularly those systems that have evolved in the absence of ungulates and their predators, are well‐documented. Yet, the many ecological outcomes of invasions, eradications, and management of island ecosystems give cause for specific investigation and reporting of results. Studying island‐endemics and the introduced species interacting with them, especially when the invaders are native to the broader regional landscape, can be informative for evolutionary biology, ecology, climate change science, and biodiversity conservation.

The influence a deer population can have in shaping its ecological environment is well‐studied, both in native habitats and on islands where they have been introduced (Potvin et al. 2003; Côté et al. 2004; Martin et al. 2009, 2011; Ramirez et al. 2012; Tanentzap et al. 2012; DiTommaso et al. 2014). Specific cases of deer herbivory affecting floristic diversity and lowering the plant species richness for a particular area have also been documented (Goetsch et al. 2011), as have the direct impacts of herbivory on certain threatened and endangered plants (Benson and Boyd 2014; Kettenring et al. 2014).

**Figure 1:**
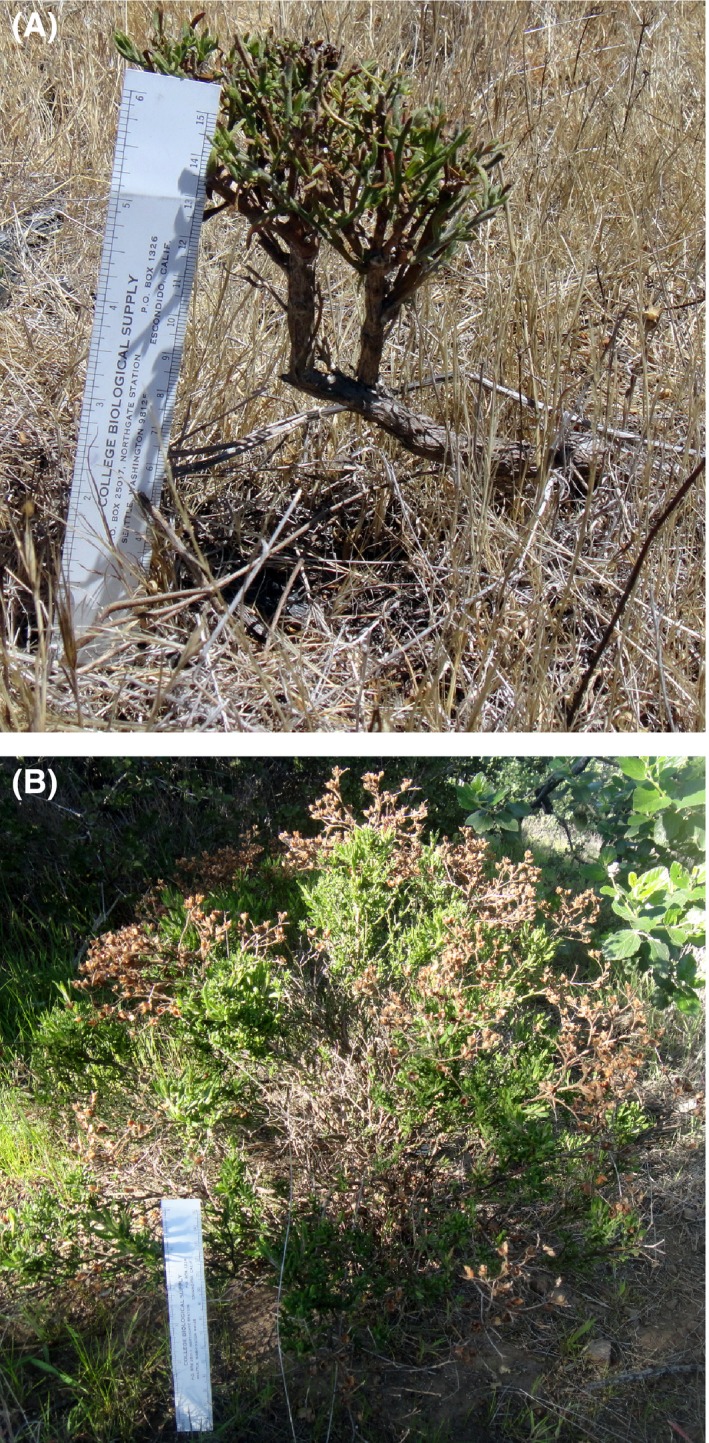
A heavily browsed individual (A) from control population C01 that failed to produce seed and a nonbrowsed individual (B) from exclosure population E03 that showed seed production success.

Introduced species are considered one of the foremost issues in conservation biology and resource management today (Pimentel et al. 2005; Simberloff et al. 2013). But, their lasting impacts on ecosystem structure and diversity, and as drivers of extinction (and perhaps speciation) are still only beginning to be understood and effectively communicated (Wilcove et al. 1998; Mooney and Cleland 2001; Lee 2002; Gurevitch and Padilla 2004; Ricciardi et al. 2013; Jeschke et al. 2014). The conditions which can make islands more susceptible to the effects of invasion (e.g., lower species richness, the novelty of invaders, competitive advantage, lack of large predators, loss of plant defenses) are well‐known concepts in island conservation and ecological theory (D'Antonio & Dudley 1995; Reaser et al. 2007). Thus, the interactions between species of conservation concern and nonnative invaders are at times played out most explicitly on islands. Many oceanic islands have evolved in isolation from large mammalian herbivores and increased susceptibility among island plants to the adverse effects of introduced herbivores has been shown (Bowen and van Vuren 1997). Are introduced mule deer negatively affecting the success of any rare plant species on Catalina? Here, we present two measures of impact to the rare, island‐endemic plant species *Crocanthemum greenei* (island rush‐rose; Fig. 1) experiencing browsing pressure from introduced mule deer (*Odocoileus hemionus*).

### Background

Native to the adjacent southern California mainland, mule deer have successfully populated Santa Catalina Island (hereafter, Catalina) after 22 individuals were first introduced in 1928–1932 (Longhurst et al. 1952). By 1949, there were approximately 2000 deer on the island (Longhurst et al. 1952), which naively equates to approximately 10 deer per km^2^ of island area. Recent population estimates (Stapp and Guttilla 2006) and the results of unpublished spotlight surveys by the Catalina Island Conservancy (conducted in July of 2012, 2013, and 2014) reflect similar densities, with estimates varying interannually from approximately 1000 to 2500 deer island‐wide. However, density is not evenly distributed throughout the island, since deer tend to concentrate in preferred habitat and are gregarious in their fine scale distributions (Stapp and Guttilla 2006).

We describe mule deer on the Catalina landscape as an extra‐local invasive species. These deer are native to the region and the particular habitat types in which they reside, but were introduced to the specific locality. This term could be useful in ecological theory when categorizing invasive species. This distinction is also important in regard to the current management actions available for controlling deer numbers on Catalina. Although clearly qualifying as an invasive species, due to the ecological situation and the deer having been a recent anthropogenic introduction, because they are a native California game species, management of the population has been working within the limits of recreational hunting seasons and state game laws. These regulations were designed to perpetuate the native natural resources and lack the effective tools (e.g., unlimited bag limits, year‐round hunting, baiting, night hunting) for reducing a deer population that would be available in the case of a more exotic invasive.

*Crocanthemum greenei* (Cistaceae) is a rare plant, precinctive to only four of the Southern California Channel Islands and is federally listed as threatened. Detailed knowledge of mechanisms influencing distribution and abundance of this sub‐shrub are mostly unknown. Although, its apparent reliance on latent populations within the soil seed‐bank has gained some clarity with the recent expansion of known occurrences following wildfire events (USFWS 2010).

Further understanding of *C. greenei* ecology is needed to inform management actions toward its recovery. It has been hypothesized that the abundance of this species has been limited by the historic abundance of introduced ungulates throughout its range. It is considered extirpated on one (San Miguel) of the four islands from which it is known. Of the remaining three (Santa Catalina, Santa Cruz, and Santa Rosa) islands with extant populations, Catalina is the only site where introduced ungulates remain today. Although past restoration efforts that eradicated feral goats (*Capra hircus*) and pigs (*Sus scrofa*) from Catalina (Schuyler et al. 2002) were likely influential actions toward a restructuring of the island's ecosystem, the presence of introduced mule deer perpetuates browsing pressure, which continues imparting an introduced top‐down selective force on the vegetative community.

Effects of the remaining introduced ungulates on *Quercus pacifica* recruitment (Manuwal and Sweitzer 2008; Stratton 2009) and overall impacts to the island ecosystem and post‐fire regeneration (Sweitzer et al. 2003; Manuwal 2007) were investigated during the subsequent years after goat and pig removal. Recently, the effects of deer browsing have been more clearly quantified regarding post‐fire regeneration of certain dominant chaparral shrubs (i.e., *Heteromeles arbutifolia*,* Rhamnus pirifolia*, and *Rhus integrifolia*); Ramirez et al. (2012) documented high levels of browser‐induced mortality for these species and suggested an increased likelihood of ecological impacts, such as vegetation‐type conversion.

While the aforementioned studies find the continued presence of introduced ungulate species on Catalina are affecting regeneration and overall structure of the common floral components of the ecosystem, a question that has not been answered quantitatively is what effect are these ungulates having, population‐wide, on a relatively rare species? Currently, it is important to confirm that not only is the overall habitat being altered by the presence of introduced ungulates, but what particular effects are being imparted on unique species. This leads to the question we addressed in this study: does the exclusion of mule deer from areas containing *C. greenei* impact size or reproductive success in the federally threatened island‐endemic plant species?

## Materials and Methods

**Figure 2:**
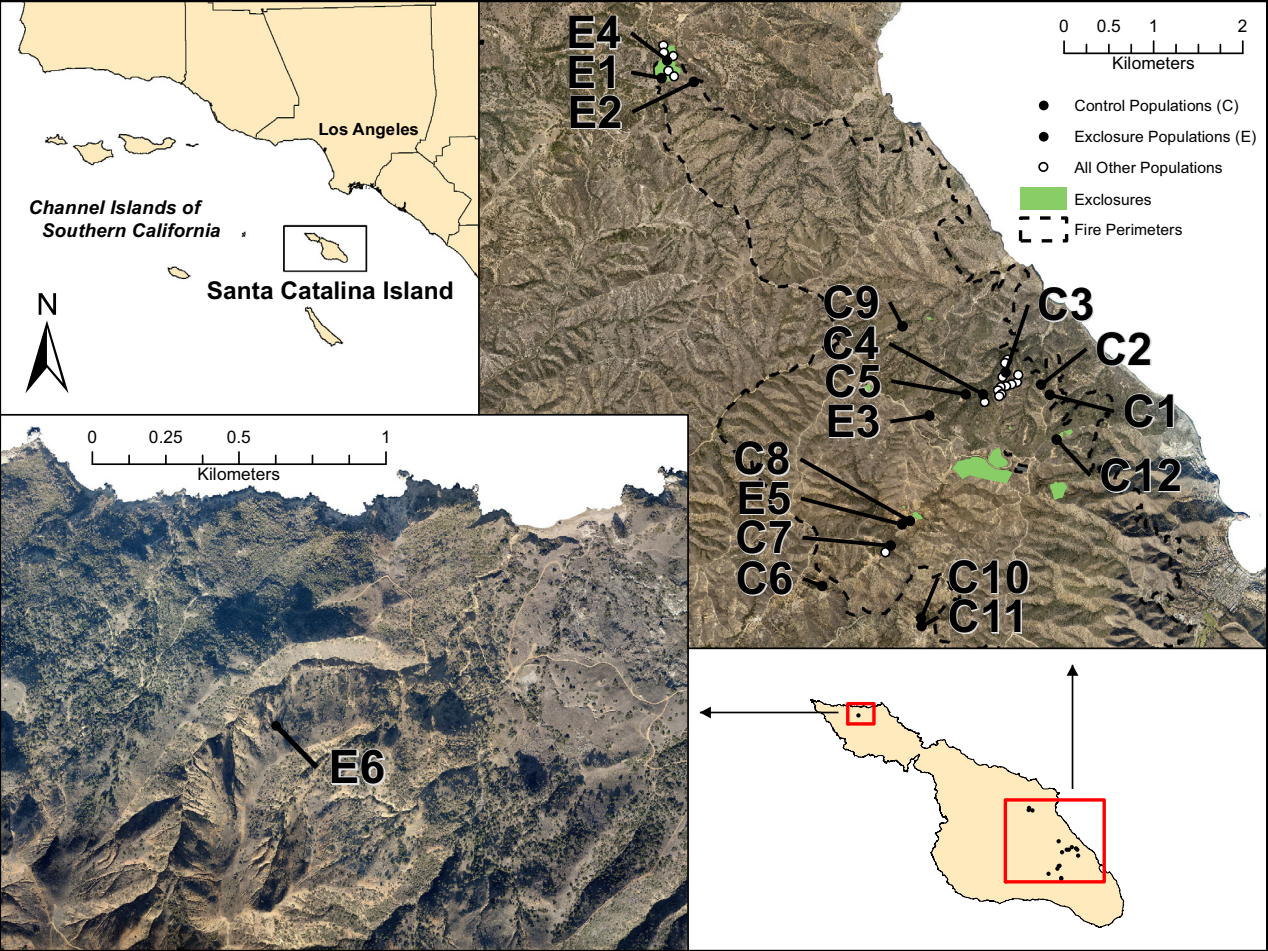
Map illustrating the locations of all known extant occurrences of *Crocanthemum greenei*, all study populations, exclosures, and the perimeters of areas burned in the 2007 fire.

Thirteen ungulate exclosures were constructed on the island over the years following an anthropogenic wildfire in 2007, which burned nearly 20‐km^2^ of the approximately 190‐km^2^ landscape (Fig. 2). Those restoration exclosures (which only exclude non‐native ungulates [deer and bison], all other wildlife can move in and out) made of eight‐foot‐tall plastic fence material (DeerBusters, Waynesboro, PA) and ranging in size from one‐fifth to 27 acres, have protected certain populations of *C. greenei* since the time of the fire. This provided our study with a high level of comparability between known age individuals that had experienced a shared disturbance and climatic regime during their life. Seventy‐five‐percent of control and 83% of exclosure populations occurred in post‐fire habitat.

At the beginning of work more directly connected to this study, four additional exclosures with sizes from less than one‐tenth to two‐fifths of an acre were built during 2010 and 2011 for the specific protection of other *C. greenei* populations on the island, three within the burned area and one at an unburned population. These extra fencing efforts ensured that our study also included control and exclosure populations from unburned habitats, although a large proportion of the *C. greenei* on Catalina is within the postfire areas and the higher percentage of our study populations being in those areas reflects that reality.

There was a 3‐year period (2010–2012) of focused searches for new *C. greenei* locations and detailed mapping of all previously known occurrences. A sample of individuals, both inside and outside of the exclosures, was selected as part of a demographic monitoring program. Exclosures had been in place from 2 to 5 years prior to sampling, essentially removing the immediate effects of browse by mule deer from the exclosure areas. We marked each individual with a unique numbered aluminum tag and logged a GPS point at sub‐meter accuracy (Trimble GeoXT) to aid in relocating the individual study plants. Over an entire season (2013), we monitored the selected *C. greenei* individuals. Our coverage of the species is representative of its overall population on the island, although there still might be isolated, undiscovered populations.

The study populations were chosen to represent all types of *C. greenei* occurrences on Catalina (i.e., different habitats, areas, densities, and population sizes). They comprise 47% of the 38 known populations on the island. Twenty individuals were sampled from each population. If the entire population consisted of <20 individuals, then all of the population was measured. Individuals were randomly chosen from each sampled population, with the one exception that visibly dead or nearly leafless and dying individuals were rejected. The measurements analyzed for stem height and seed production discussed in this paper are of the adult study individuals only and all were living and capable of reproduction during the measurement season.

For a total of 298 individuals across 18 populations of *C. greenei* on Catalina, we measured the stem and recorded annual seed production success of each individual. Samples were measured from 12 control populations of individuals not protected by exclosure fencing (*n *=* *203) and six treatment populations of individuals from within the exclosure areas (*n *=* *95).

Stem was measured as the length (cm) from base of a main stem at ground level to tallest point (excluding the inflorescence if present). The same researcher (A.E.C.) measured all individuals in the study, which ensured data was generated from a consistent technique. During the monitoring year, reproductive status for each individual was recorded in a simple binary manner: if an individual (*x*_i_) exhibited any seed production (*x*_i_ = 1), or if it did not (*x*_i_ = 0).

True population measurements of the stems and documentation of seed production success were attained for all individuals within five control populations (C02, C05, C08, C09, C11) and three exclosure populations (E01, E03, E04).

### Statistical analyses

For inferential stem measurement analysis, we used a linear mixed effects model (LMM) fit by maximum likelihood estimation. Our data were sufficient in regard to all model assumptions. We calculated Morans's I from a distance matrix of the population coordinates and determined that mean population measurements exhibited spatial autocorrelation. We then incorporated all sample measurements in evaluating spatial model structures against a null model, leaving out our fixed effect. Lowest AIC (Akaike information criterion) value determined the best‐fit model. A spherical model provided the best fit for our spatial data and the associated correlation structure was incorporated into our LMM. Stem measurement was modeled as a function of the fixed effect of exclusion (i.e., control or exclosure). Population was considered as a random effect, because individuals were measured across a selection of distinct populations, and we were not able to set up both control and exclosure sampling for each population, or each area of the island in which the species occurs.

For seed production success, there was complete separation in the data between populations. It was our assessment, through field observations within exclosures, that perfect seed production success in a population is often true for *C. greenei*. Additionally, we often observed that nearby individuals outside of those exclosures failed to produce any seed when heavily browsed. The three control populations where true complete failure was measured (C02, C05, C08) and three exclosure populations where true complete success was measured (E01, E03, E04) further support this assumption. As a result, we did not attempt to address the complete separation in our data through modeling (e.g., with penalized logistic regression). It follows that we did not model seed production, as we did with stem measurements, to generate a comparison between control and exclosure individuals as a whole. Therefore, only comparisons at the population level were done and we did not calculate an overall effect of deer exclusion on seed production success for *C. greenei*.

We used binomial exact tests to analyze seed production success. All exclosure populations showed complete success. Control populations were tested against the lower limit of the 99.5% confidence intervals among the sample exclosure populations. In other words, significance was measured against the exclosure population with the least evidence for complete success: 19 successes out of 19 samples (E06, 99.5% CI: 0.73–1.00). Confidence interval and *P*‐value significance level (0.005) was adjusted from 0.05 for multiple comparisons using a Bonferroni correction for the 10 separate binomial exact tests.

We checked assumptions and conducted all analyses and graphing in the R environment (R Core Team 2014) using the packages *ape* (Paradis et al. 2004), *geoR* (Diggle and Ribeiro 2007), *ggplot2* (Wickham 2009), and *nlme* (Pinheiro et al. 2014).

## Results

**Figure 3:**
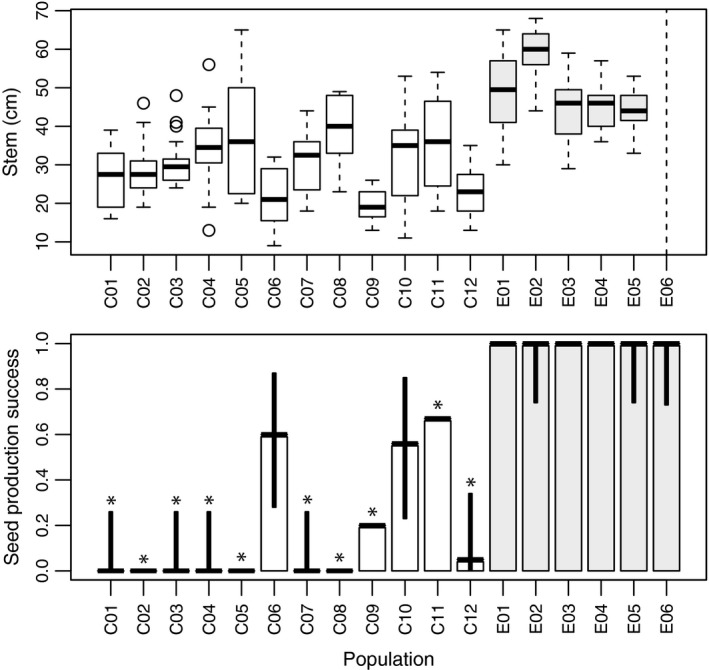
Data summarized by population for stem measurements (top plot) and seed production success (bottom plot). Stem measurement is illustrated with standard box plots. Seed production success is graphed with bars that include vertical error lines for the 99.5% CI of each population's proportion of success, based on binomial exact tests. Asterisks indicate significantly less successful control populations.

Among the populations of *C. greenei* observed during our study, deer exclusion significantly affected stem height (LMM: df 15, *t* 3.78, *P *=* *0.0018), increasing it on average by 18.8 cm (SE = 4.97). Stem measurements have been plotted by population to be viewed along with seed production success data (Fig. 3; see also: descriptive statistics in Table S1A).

Exclosure populations exhibited complete seed production success, whereas control populations showed lower success and exhibited complete failure within 58% of the populations (Fig. 3). Of the sampled control populations, five of seven were significantly (*P *<* *0.005) less successful than the least successful exclosure population. Populations C01, C03, C04, and C07 all showed complete failure with 20 sample individuals each (0/20 = 0.00, 99.55% CI: 0.00–0.26, *P *=* *4.24 × 10^−12^). All control populations in which we measured true success through measurement of every individual (C02, C05, C08, C09, C11) were less successful than exclosure populations (Fig. 3; see also: descriptive statistics in Table S1B).

## Discussion

We aimed to quantify the effect of ungulate exclusion in a way that would represent the current extant range of *C. greenei* on Catalina. Through thoroughly searching the island for occurrences, we ensured that the full range of the Catalina populations were given coverage. We expected that there would be some variability between populations (i.e., site conditions for growth and susceptibility to browse, as well as differences in local deer abundance) that needed to be accounted for with a large, spatially diverse sample.

With the large sample of individuals in the study and the effort required to measure accurately how much seed each plant was producing, we did not attach a quantitative metric for the actual amount of seed produced per individual. Given our field observations during the years preceding the sampling, the majority of browsed individuals experienced complete annual failure and very few unprotected individuals fell into the measurable range of partial success. We do not ignore the fact that there are complex variables involved in the longer‐term outcome of seed production (e.g., difficulty comparing unbrowsed profusely seeding individuals that may live shorter lives to less annually productive but possibly longer living browsed individuals [Aragón et al. 2009]) and seed‐bank dynamics (Maron and Gardner 2000; Maron and Crone 2006). Those questions are beyond the scope of this 1‐year dataset, but they may also be undermined by the severity of impact and the life history disruption being caused if the level of herbivory present continues over the long‐term. Since the impacts documented here involved a high percentage of total success and failure, the impact of browsers on seed production for individuals of this species were sufficiently measured by the success‐failure analysis we employed. If impacts were lessened (e.g., in the case of lower deer densities) and most individuals were able to produce some seed each season, then a more tedious seed counting approach would be necessary to assess the effect of deer removal.

Stem measurement data also implies less reproductive capacity among control individuals. In general, when the smaller browsed plants did produce seed, field notes associated with the data for the control group described poor productivity (e.g., “severely browsed, one flower;” “only one branch fruiting”). The significant reduction in stem height that was detected might reflect a reduction in potential capacity for seed production, when and if control individuals are intermittently released from browsing to produce seed. It follows that exclosure individuals that experience complete seed production success also experience a compounding effect of increased seed production due to increased stem size. A conservative aspect of our binary seed production data is that since all a browsed individual needed was one flower to remain and produce seed for it to have the same statistical weight as an exclosure individual that seeded profusely, our methods imply that seed production was increased more substantially due to exclosures than we can report based on the data.

It is notable that, when stem measurements are plotted, there are some larger outliers among the control individuals (Fig. 3, populations C02, C03, C04) and all had accompanying field notes within the dataset describing natural protection from browse by surrounding shrubs, branches, or physical topography. Considering that deer were effectively excluded from browsing these individuals, the heights of these individuals do not detract from our conclusions, although they do minimize the effect we detected. The site‐specific natural protection from browse may play a small role in the ability of the species to successfully coexist with introduced browsers. Although, *C. greenei* is a plant that grows in areas with little surrounding cover and prefers full exposure to sunlight, conditions which generally expose it fully to browsing by ungulates. Being a small subshrub, it also cannot grow above the reach of browsers. Therefore, this effect is minimal.

The effect of excluding introduced ungulates on the biological success of this particular rare plant species has been quantified for the first time. This initial data may eventually be extrapolated when evaluating the future persistence of specific populations or the success of the species as a whole on Catalina. Our study is also a strong indication that current deer management strategies on Catalina are not sufficient to protect this island‐endemic plant species. Evidence such as this, of a threatened species and the effects of a given threat, remain valuable in conservation biology and ecology. In the long‐term, we recommend continued monitoring of the outcomes for *C. greenei* populations, both exposed to and protected from herbivory. Knowledge of management outcomes and studies in the ecology of plant rarity can benefit from continued work on the distribution of this rare plant species on Catalina. This work could be framed with respect to future changes in browser density, habitat, climate, and disturbance such as fire and erosion. Additionally, our field observations indicate it has higher palatability to deer than the more common and widespread congeneric (*Crocanthemum scoparium* [peak rush‐rose]) with which it co‐occurs on the islands. *Crocanthemum scoparium* does not appear to be browsed by deer much. We speculate based on these anecdotal observations that this may suggest a lack of defenses against browsing pressure due to *C. greenei*'s evolution on islands free from ungulate browsers. Lending support to this hypothesis, the more common *C. scoparium* also occurs in cismontane mainland California (CCH 2014), which is within the native range of mule deer and other large ungulates. This may be an opportunity for evolutionary biology research with this genus on the Channel Islands.

It is important to precisely classify invaders when reporting their effects. This interaction between an introduced extra‐local invader and an island‐endemic species that likely evolved in its absence may be an example of a plant species evolving without defenses against ungulate herbivores. The quantification of specific interactions provides valuable information for conservation‐focused land managers. This approach may also help inform or guide broader research involving abrupt species range shifts, which go beyond the realm of island biogeography and our particular research. We encourage continued efforts to collect specific information that can empirically underpin theories in ecology and invasion science, where it has become clear that the effects of introduced or invading species are not easily generalized.

## Conflict of Interest

None declared.

## Supporting information


**Table S1**. (A) The descriptive statistics for stem measurements for all populations. (B) The measurements of seed production success for all study populations.
