# Supplementary material for: Exclusion of introduced deer increases size and seed production success in an island‐endemic plant species
Source: Ecol Evol. 2016 Jan 9;6(2):544–51. doi: 10.1002/ece3.1885 (PMC4729259; doi:10.1002/ece3.1885)
Supplement: Supplementary file 1 — Table S1. (A) The descriptive statistics for stem measurements for all populations. (B) The measurements of seed production success for all study populations. [file ECE3-6-544-s001.docx]

**Table S1A. The descriptive statistics for stem measurements for all populations. Asterisks denote populations in which every individual was measured. Standard deviations are presented as sample (**$\boldsymbol{s}$**) or population (**$\boldsymbol{\sigma}$**) values.**

| **Population** | ***N*** | **Range** | **Median** | **Mean** | $\boldsymbol{s}$ | $\boldsymbol{\sigma}$ |
| --- | --- | --- | --- | --- | --- | --- |
| C01 | 20 | 16 – 39 | 27.5 | 27.3 | 7.6 |  |
| C02* | 18 | 19 – 46 | 27.5 | 28.6 |  | 6.5 |
| C03 | 20 | 24 – 48 | 29.5 | 30.7 | 6.3 |  |
| C04 | 20 | 13 – 56 | 34.5 | 34.1 | 9.6 |  |
| C05* | 11 | 20 – 65 | 36.0 | 37.5 |  | 14.8 |
| C06 | 20 | 9 – 32 | 21.0 | 21.9 | 7.6 |  |
| C07 | 20 | 18 – 44 | 32.5 | 30.5 | 7.5 |  |
| C08* | 6 | 23 – 49 | 40.0 | 38.3 |  | 9.2 |
| C09* | 15 | 13 – 26 | 19.0 | 19.9 |  | 4.0 |
| C10 | 18 | 11 – 53 | 35.0 | 33.7 | 11.8 |  |
| C11* | 15 | 18 – 54 | 36.0 | 35.8 |  | 12.0 |
| C12 | 20 | 13 – 35 | 23.0 | 22.9 | 6.4 |  |
|  |  |  |  |  |  |  |
| E01* | 16 | 30 – 65 | 49.5 | 48.5 |  | 10.7 |
| E02 | 20 | 44 – 68 | 60.0 | 59.3 | 6.4 |  |
| E03* | 11 | 29 – 59 | 46.0 | 43.8 |  | 8.6 |
| E04* | 9 | 36 – 57 | 46.0 | 45.7 |  | 6.2 |
| E05 | 20 | 33 – 53 | 44.0 | 44.3 | 5.5 |  |

**Table S1B. The measurements of seed production success for all study populations. Asterisks denote populations in which every individual was measured, providing the true success. Confidence intervals and significance were calculated using binomial exact tests. P-values are with respect to the null hypothesis that a control population’s true probability was 0.73 (i.e., within the range of our least confident measurement of complete success among exclosure populations).**

| **Population** | **Successes/Total** | **Probability** | **99.5% CI** | **P-value** |
| --- | --- | --- | --- | --- |
| **C01** | 00/20 | 0.00 | 0.00 – 0.26 | $4.24\times{10}^{-12}$ |
| **C02*** | 00/18 | 0.00 |  |  |
| **C03** | 00/20 | 0.00 | 0.00 – 0.26 | $4.24\times{10}^{-12}$ |
| **C04** | 00/20 | 0.00 | 0.00 – 0.26 | $4.24\times{10}^{-12}$ |
| **C05*** | 00/11 | 0.00 |  |  |
| C06 | 12/20 | 0.60 | 0.28 – 0.87 | 0.21 |
| **C07** | 00/20 | 0.00 | 0.00 – 0.26 | $4.24\times{10}^{-12}$ |
| **C08*** | 00/06 | 0.00 |  |  |
| **C09*** | 03/15 | 0.20 |  |  |
| C10 | 10/18 | 0.56 | 0.23 – 0.85 | 0.11 |
| **C11*** | 10/15 | 0.67 |  |  |
| **C12** | 01/20 | 0.05 | 0.0001 – 0.34 | $2.34\times{10}^{-10}$ |
|  |  |  |  |  |
| E01* | 16/16 | 1.00 |  |  |
| E02 | 20/20 | 1.00 | 0.74 – 1.00 |  |
| E03* | 11/11 | 1.00 |  |  |
| E04* | 09/09 | 1.00 |  |  |
| E05 | 20/20 | 1.00 | 0.74 – 1.00 |  |
| E06 | 19/19 | 1.00 | 0.73 – 1.00 |  |
